# Supplementary material for: Bridging the Methodological Gap Between Inertial Sensors and Optical Motion Capture: Deep Learning as the Path to Accurate Joint Kinematic Modelling Using Inertial Sensors
Source: Sensors (Basel). 2025 Sep 14;25(18):5728. doi: 10.3390/s25185728 (PMC12473578; doi:10.3390/s25185728)

Supplement Information:

Supplement notes 1: Custom Biomechanical Loss Function.

This document details the mathematical equations for the custom biomechanical loss function (`custom_biomech_loss`). The loss combines multiple components to ensure accurate, consistent, and biomechanically plausible predictions for sequential data.

General Mean Squared Error (MSE)

The general MSE term computes the average squared error across all time steps (t), spatial dimensions (s), and features (f).

$$MSE_{general} = \left(1/(T * S * F)\right) * \Sigma_{t=1}^{T} \Sigma_{s=1}^{S} \Sigma_{f=1}^{F} \left(y_{true}[t, s, f] - y_{pred}[t, s, f]\right)^2$$

Foot-Specific MSEs

The loss emphasizes key features for the right and left feet:

$$MSE_{Rfoot} = \left(1/\left(T * S * \left|F_{Rfoot}\right|\right)\right) * \Sigma_{t=1}^{T} \Sigma_{s=1}^{S} \Sigma_{f \in F_R foot} \left(y_{true}[t, s, f] - y_{pred}[t, s, f]\right)^2$$

$$MSE_{Lfoot} = \left(1/\left(T * S * \left|F_{Lfoot}\right|\right)\right) * \Sigma_{t=1}^{T} \Sigma_{s=1}^{S} \Sigma_{f \in F_L foot} \left(y_{true}[t, s, f] - y_{pred}[t, s, f]\right)^2$$

Prediction Restriction Loss

This component ensures temporal consistency by penalizing discrepancies in sequential changes over multiple time-step lags (k = 1, 2, ..., 5):

$$\Delta_k y_{true} = y_{true}[k, :, :] - y_{true}[-\mathrm{k}, :, :]$$

$$\Delta_k y_{pred} = y_{pred}[k, :, :] - y_{pred}[-\mathrm{k}, :, :]$$

$$MSE_{restric} = \Sigma_{k=1}^{5} \left(1/(T - k)\right) * \Sigma_{t=1}^{T-k} \Sigma_{s=1}^{S} \Sigma_{f=1}^{F} \left(\Delta_k y_{true}[t, s, f] - \Delta_k y_{pred}[t, s, f]\right)^2$$

Top 24 Loss MSE

The loss prioritizes the 24 features with the largest prediction errors. First, feature-wise, MSEs are computed and sorted in descending order. Then, the top 24 are averaged:

$$MSE_f = \left(1/(T * S)\right) * \Sigma_{t=1}^{T} \Sigma_{s=1}^{S} \left(y_{true}[t, s, f] - y_{pred}[t, s, f]\right)^2$$

$$MSE_{top24} = (1/24) * \Sigma_{f=1}^{24} MSE_f, top$$

Final Loss Combination

The final loss is a weighted combination of all components, with greater emphasis on temporal consistency and biomechanical priorities:

$$Loss = sqrt\left(2 * \left(MSE_{general} + MSE_{Rfoot} + MSE_{Lfoot}\right) + 4 * MSE_{restric} + MSE_{top24}\right)$$

Supplement Tables

Supplement Table 1. Average Prediction error of markers predicted marker positions against optical motion capture (OMC) marker positions at initial validation in our data presented in centimetres (cm) when a general mean square error loss function was used for training of the model

**Definition**	Markers	X (cm)	Y (cm)	Z (cm)
Left ASIS	LASI	2.5 ± 1.0	2.8 ± 0.8	4.3 ± 2.7
Right ASIS	RASI	5.3 ± 12.3	7.4 ± 17.1	12.6 ± 35.8
Left PSIS	LPSI	6.7 ± 17.2	8.5 ± 20.9	12.9 ± 36.8
Right PSIS	RPSI	2.7 ± 1.2	3.7 ± 1.4	3.7 ± 2.0
Left thigh	LTHI	2.5 ± 1.0	3.7 ± 1.4	5.5 ± 2.7
Left knee	LKNE	2.9 ± 1.2	4.2 ± 1.0	3.6 ± 1.2
Left tibia	LTIB	2.9 ± 1.1	4.7 ± 1.3	3.6 ± 2.0
Left ankle	LANK	3.1 ± 1.1	5.1 ± 2.2	2.6 ± 0.9
Left heel	LHEE	2.7 ± 0.7	5.1 ± 2.4	2.9 ± 1.1
Left toe	LTOE	3.0 ± 0.7	5.5 ± 2.9	2.2 ± 0.8
Right thigh	RTHI	2.5 ± 1.1	4.3 ± 1.2	6.3 ± 3.8
Right knee	RKNE	2.5 ± 1.1	4.3 ± 1.4	3.4 ± 2.0
Right tibia	RTIB	2.4 ± 1.1	4.8 ± 2.3	3.3 ± 1.8
Right ankle	RANK	2.5 ± 0.7	5.3 ± 3.1	3.1 ± 1.2
Right heel	RHEE	6.3 ± 14.3	10.4 ± 21.3	7.4 ± 17.3
Right toe	RTOE	2.8 ± 1.4	6.1 ± 3.7	2.3 ± 0.6
Average All Marker		3.3 ± 3.6	5.4 ± 5.3	5.0 ± 7.0

Notes: ASIS = Anterior superior iliac spine, PSIS = Posterior superior iliac spine

Supplement Table 2. Joint angle comparison between optical motion capture (OMC) and predicted markers position computed joint angles using our proposed method when a general mean square error loss function was used for training of the model

Datasets	Same dataset
Joints	Non-DTW
Right Hip	19.5 ± 33.0
Right Knee	27.4 ± 69.6
Right Ankle	11.1 ± 3.7
Right Hip	14.7 ± 27.4
Right Knee	15.8 ± 10.0
Right Ankle	10.8 ± 7.7

All values represent root mean squared error (RMSE) in degrees.Non-DTW = no alignment of data using Dynamic time warping

Supplement Table 3. Comparison of joint angle prediction in the current study with past studies.

Article	Deep learning model	Participants	Input data	sensor combination	Compared Against	Prediction task	Data Split	RMSE
[16]	CNN	10 healthy	Acc, AV (raw + Simulated)	multi	OMC	H+K+A flex	sub-wise	2-8
[20]	FF	8 healthy	Acc, AV	multi	OMC	H+K+A flex	Sub-wise	MAD = 7.13
							Random	MAD = 1.98
[22]	LSTM	30 healthy	Acc, AV and Euler angle	single	OMC	H+K+A flex	sub-wise	4.35-7
							Random	3.06-5.76
							within sub	0.14-0.47
[39]	FF	23 real, 93 simulated	Acc, AV	multi	OMC	all	sub-wise	2 -15
[40]	CNN+LSTM	420 walking and 580 running	Simulated Acc, AV	multi	OMC	all	Random	2.13-6.55
[41]	FF	8 healthy	IMU orientation	multi	OMC	H+K+A flex	Random	2.0-9.0
[18]	LSTM	18 healthy	Acc, AV	Two	OMC	H+K+A flex	sub-wise	2.0-6.0
Current Study	LSTM + Attention layer	18 healthy	Acc, AV	multi	OMC	H+K+A flex	sub-wise	2.0-7.0

Note:
Models: FF- forward feed, LSTM- long short-term memory, CNN- Convolutional neural network
Input data: Acc- 3D Acceleration, AV: 3D Angular velocity, raw- collected with IMUs, simulated: synthetic data simulated from OMC data
Compared against: OMC: Optical motion capture system,
Prediction task: H- hip joint, K- knee joint, A- Ankle joint, flex- flexion angle, all – all lower body joint angles
Data split: Sub-wise: subject-wise splitting, Random: random splitting technique, within sub: single subject testing

Supplement Figures:

Supplement Figure 1. Deep learning model architecture. An encoder-decoder type architecture consists of convolution, long short-term memory, attention, and forward-feed layers. The input of the model was n (number of samples) x 101 (normalized gait cycle) x 14 (7 sensor x 2 acceleration and gyroscope data) x 4 (X, Y, Z and Normalized Magnitude). The output of the model was n (number of samples) x 101 (normalized gait cycle) x 16 (total number of markers) x 3 (marker position in X, Y and Z direction).

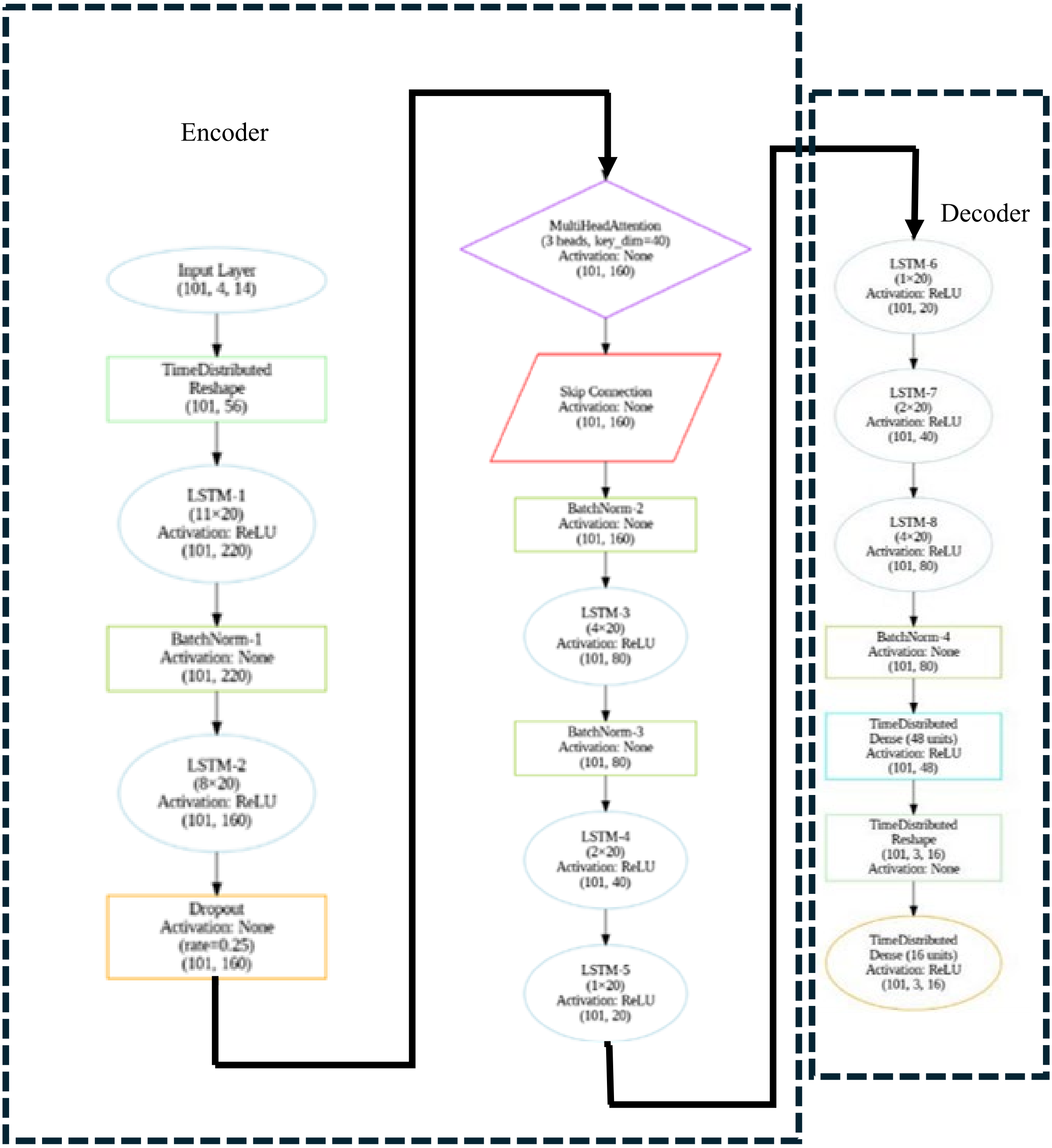

Supplement Figure 2. Mean joint angle plots of a single participant for optical motion capture (OMC) vs inertial measurement unit (IMU)-based approach. The predictions shown are not aligned with dynamic time warping, and this shows predictions when a general loss function of mean square error is used to train the model. High deviations are visible in the blue predicted marker-based joint angles, which was the reason for introducing the custom loss function.

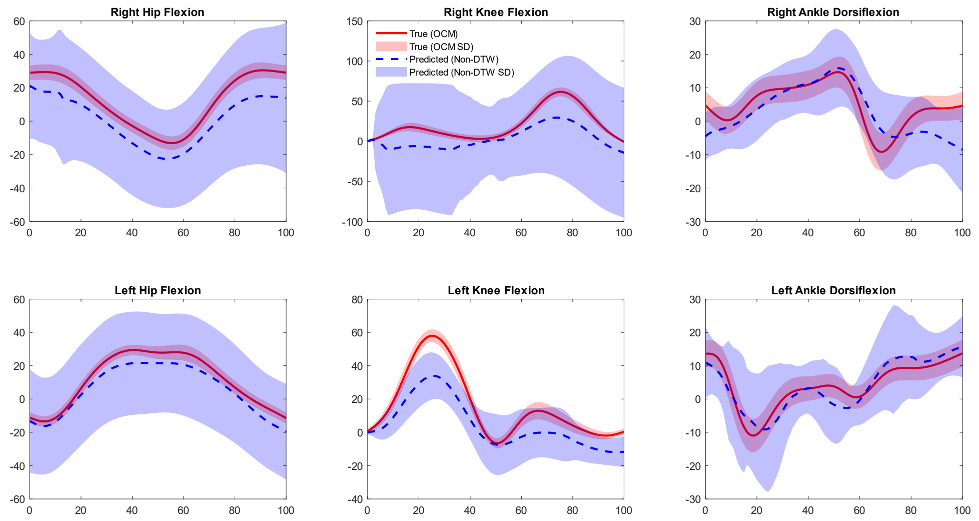

Supplement Figure 3: Bland–Altman plots comparing predicted and true joint angles for six lower-limb joints during gait: right hip flexion, right knee flexion, right ankle dorsiflexion, left hip flexion, left knee flexion, and left ankle dorsiflexion. The plots show the difference between predicted and true joint angles (y-axis) against the mean joint angle (x-axis). The red line represents the mean bias, and the dashed blue lines indicate the upper and lower limits of agreement (LoA).

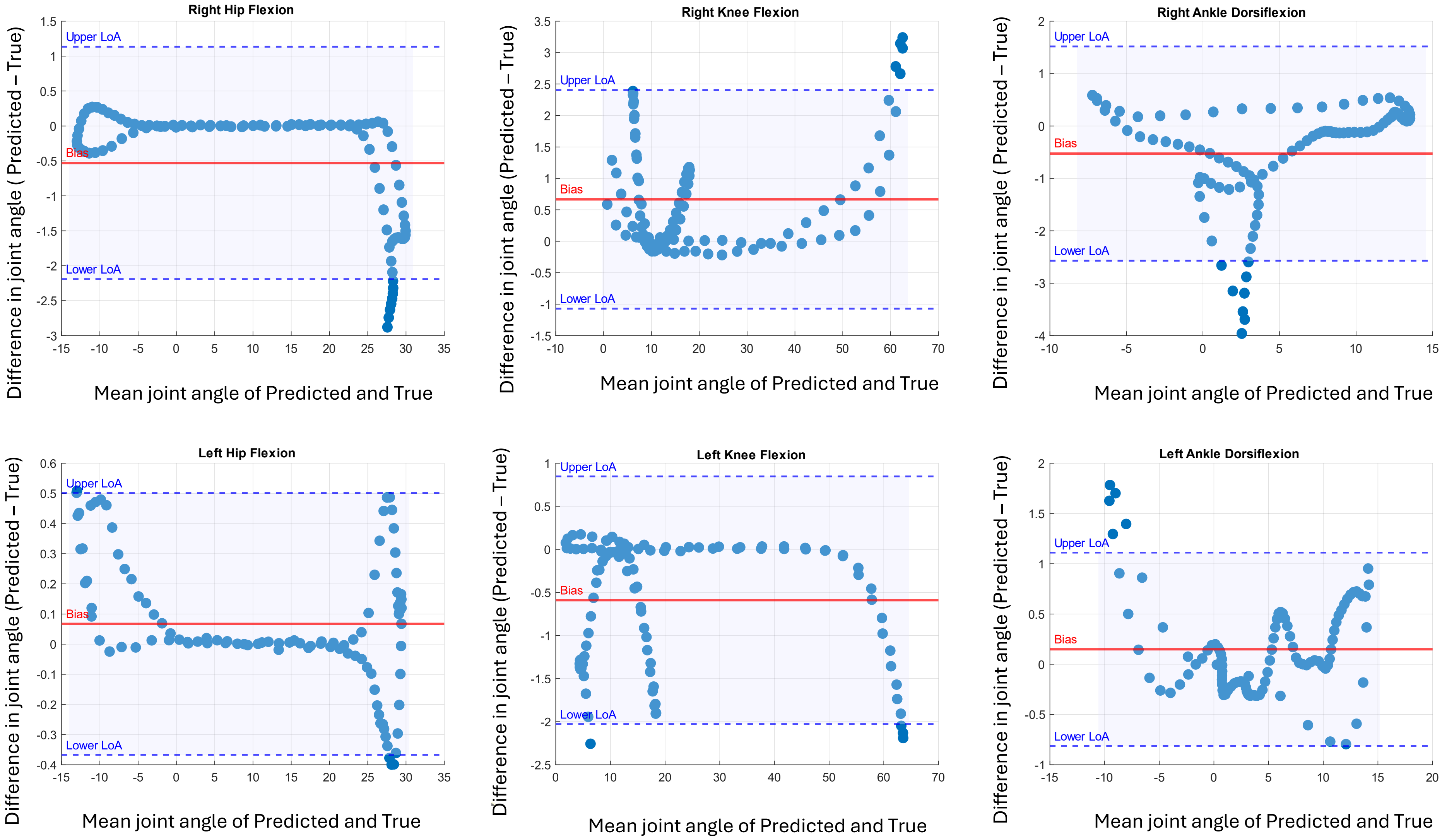

Supplement Figure 4. Joint angle plots from wanderman et al. [24] external data for a single participant. Here, shank and foot sensor locations were switched to check if the model does not produce gait cycle-like data for arbitrary inputs. This suggested that the model does not produce gait cycle-like data for arbitrary inputs.

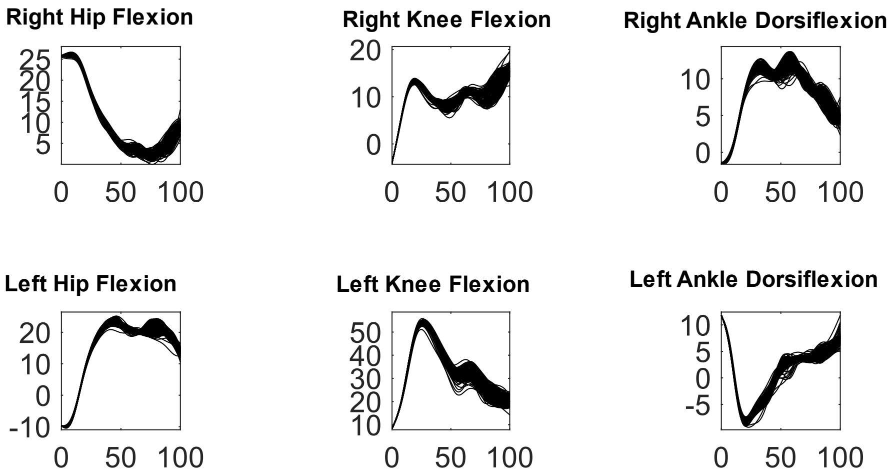

Supplement: Supplementary file 1 [file sensors-25-05728-s001.zip › sensors-3835676-supplementary.pdf]
