# Supplementary figures and images for: Bridging the Methodological Gap Between Inertial Sensors and Optical Motion Capture: Deep Learning as the Path to Accurate Joint Kinematic Modelling Using Inertial Sensors
Source: Sensors (Basel). 2025 Sep 14;25(18):5728. doi: 10.3390/s25185728 (PMC12473578; doi:10.3390/s25185728)

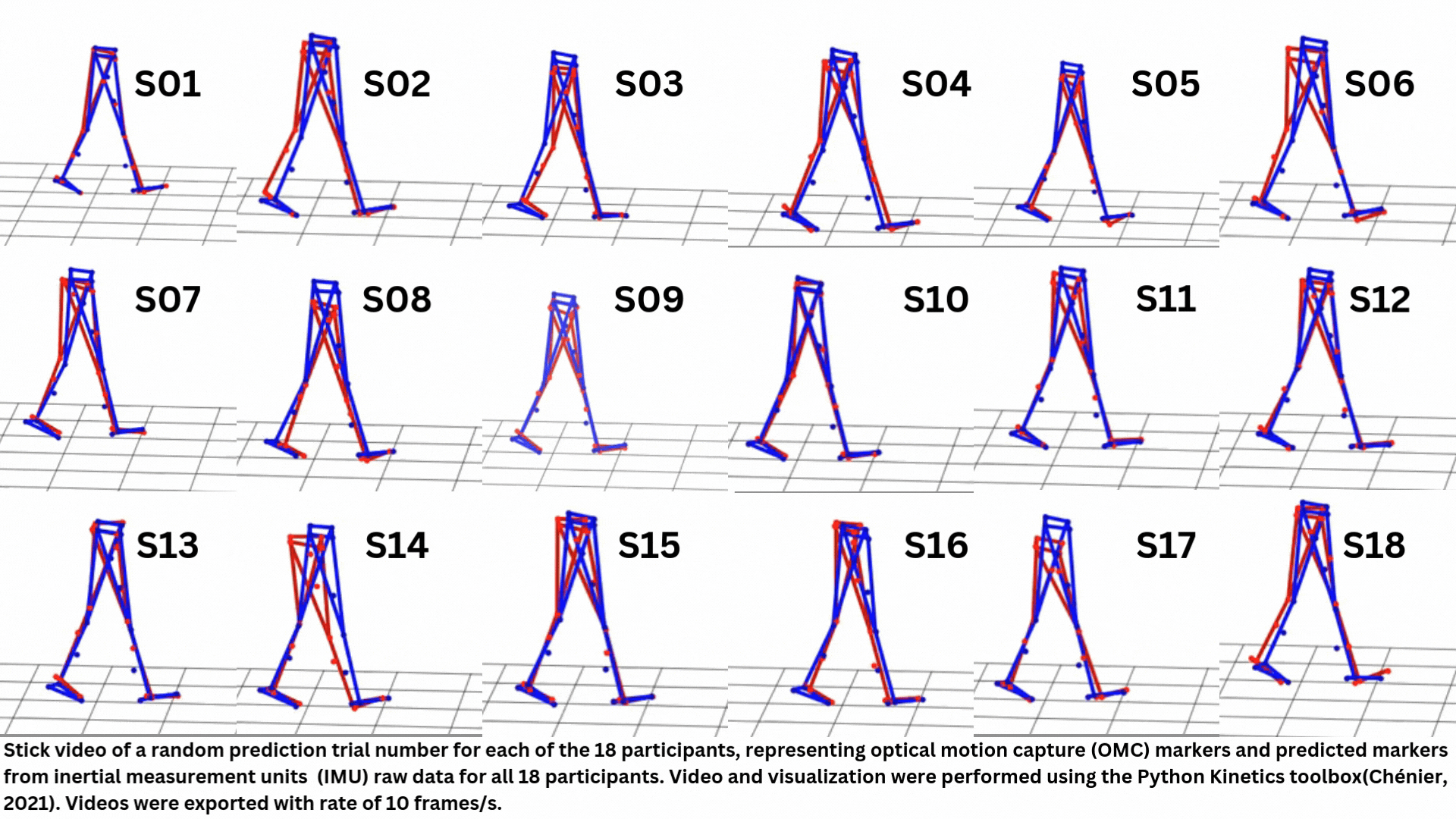

Supplement: Supplementary file 1 [file sensors-25-05728-s001.zip › sensors-3835676.gif]
